# Tobacco and the Soldier

**Published:** 1916-02-05

**Authors:** 


### TOBACCO AND THE SOLDIER.

### A Medico-Military Problem.

The newspaper controversy which has been carried on lately between those who would like to see all civilians give up tobacco as a war economy, and those who do not agree with this particular form of self-discipline, has left on one side the medical aspects of the soldiers' tobacco. It is true that the possible supplanting of the cigarette and the cheroot among affluent smokers by the pipe has been suggested as a conceivable outcome of the war; but we think the wish was father to the thought in the mind of the journalist who made some amusing "copy" out of the idea. At any rate the cigarette still retains an overwhelming preponderance in popularity over all other forms of tobacco with the men in the ranks.

It is, however, not with the social or ethical aspects of tobacco-smoking that the Army doctors are concerned: they have enough to do with the medical results, though these in turn, since they are usually the result of smoking to excess, are closely interwoven with social, moral, and economic factors. When the campaign was still quite young—that is to say, before the end of 1914—it was found that a number of men were being invalided from the Front, and were arriving in England *via* the Bases, with diagnoses of various forms of cardiac mischief, chiefly valvular disease. It was further found that the majority of these men, on careful examination, displayed no signs of valvular disease whatever; in fact, they had been misdiagnosed. In passing, it is right to explain that diagnosis is no easy matter at the Front: imagine a medical officer anywhere within five miles of the trenches trying to listen to the heart sounds with artillery fire assailing his ears from all directions.

### Smoker's Heart.

Well, the result of investigation of these cases showed that the great majority of them were examples of various degrees of cardiac dilatation and debility due to excessive smoking, or what is usually termed "smoker's heart." A memorandum embodying this experience was duly drawn up and dispatched to France for distribution to those whom it might concern; and if there were afterwards a certain number of misdiagnosed cases, at least they were very much fewer than before. As instances of the abuse of tobacco which the British soldier will practise when he gets the chance, the following personal experiences of the writer will suffice. A soldier arrived at a base hospital with symptoms ascribable to a disordered heart. After careful examination the conclusion was reached that the heart was quite healthy, but was suffering from the toxic effects of too much tobacco: the man's fingers were stained dark brown. Ensued a dialogue much to this effect: "Your heart's all right, Smith; but you've been smoking much too heavily, and you must drop it." "I'm a fairly moderate smoker, sir." "What do you call moderate? Fifty cigarettes a day, I suppose?" "About eighty, sir, most days." The man got speedily well when deprived of his poison, and soon went back to his regiment. On another occasion a soldier wrote to his wife that she need not send him any tobacco for a while, as he had got two pounds of it and also five hundred cigarettes, distributed to him free from various sources. A boy of eighteen was in hospital for a (medical) illness which caused a certain amount of anxiety; as part of the treatment he was deprived of tobacco, to which he was addicted. On three occasions he managed to secure some and smoke it surreptitiously at night: on each occasion he was betrayed by the state of his heart next morning, which had perceptibly dilated and was beating at more than twice the previous rate.

### Excessive Smoking in the Trenches.

It may be taken as undeniable that the British Army smokes more than is good for its health; but the remedy is not easy to propound. Officers and men in the trenches cannot be deprived of one of their main solaces, even though they do abuse it and, in a percentage of cases, reduce their military efficiency thereby. As long as private funds are permitted to send out cigarettes and tobacco to the troops little control can be maintained over their distribution; and even if strict supervision over all Government and other free issues were established, the private supplies from home would remain unaffected. It is quite useless to preach moderation, either to officers or men, for health reasons. They take no notice of medical sermons, and few medical officers like to deliver them. But if, as seems very probable, our soldiers return after the war with their craving for tobacco multiplied manifold, the time will then have come for such swingeing taxation, in the writer's opinion, as will compel the immoderate smoker to consider his pocket as well as his palate.

